# Alternate Splicing of Interleukin-1 Receptor Type II (IL1R2) *In Vitro* Correlates with Clinical Glucocorticoid Responsiveness in Patients with AIED

**DOI:** 10.1371/journal.pone.0005293

**Published:** 2009-04-29

**Authors:** Andrea Vambutas, James DeVoti, Elliot Goldofsky, Michael Gordon, Martin Lesser, Vincent Bonagura

**Affiliations:** 1 The Apelian Cochlear Implant Center, Department of Otolaryngology, North Shore-LIJ Health System, Clinical Teaching Campus for the Albert Einstein College of Medicine, New Hyde Park, New York, United States of America; 2 The Feinstein Institute for Medical Research, Manhasset, New York, United States of America; Massachusetts General Hospital/Harvard University, United States of America

## Abstract

Autoimmune Inner Ear Disease (AIED) is poorly characterized clinically, with no definitive laboratory test. All patients suspected of having AIED are given glucocorticoids during periods of acute hearing loss, however, only half initially respond, and still fewer respond over time.

We hypothesized that AIED is a systemic autoimmune disease characterized by dysfunctional peripheral blood mononuclear cells (PBMC) responses to a unique cochlear antigen(s). To test this hypothesis, we examined end-stage AIED patients undergoing cochlear implant surgery and compared autologous perilymph stimulated PBMC from AIED patients to controls. We determined that autologous perilymph from AIED patients was unable to induce expression of a long membrane-bound Interleukin-1 Receptor Type II (mIL1R2) transcript in PBMC as compared with controls, despite similar expression of the short soluble IL1R2 (sIL1R2) transcript (p<0.05). IL1R2 is a molecular decoy that traps interleukin-1β (IL-1β) and does not initiate subsequent signaling events, thereby suppressing an inflammatory response. IL1R2 transcript length is regulated by alternate splicing, and the major inhibitory function is attributed to the full-length mIL1R2. In addition, IL1R2 expression is induced by dexamethasone.

Separately, we prospectively examined patients with newer onset glucocorticoid-responsive AIED. Immediately prior to clinical treatment for acute deterioration of hearing thresholds, their PBMC demonstrated a robust induction of mIL1R2 in PBMC in response to dexamethasone *in vitro* that correlated with a clinical response to prednisone *in vivo* (p<0.0001) as measured by hearing restoration. In contrast, clinically steroid unresponsive patients demonstrated high basal levels of mIL1R2 in their PBMC and only minimally augmented expression in response to dexamethasone. Thus, induced expression of mIL1R2 appears to be a protective mechanism in hearing homeostasis and warrants further investigation in a large prospective clinical trial to determine if IL1R2 can be used as a specific biomarker for AIED.

## Introduction

Sensorineural hearing loss (SNHL) is a common otologic problem that typically is not amenable to medical intervention. Potentially reversible SNHL can be divided into two sub-groups: (1) Autoimmune Inner Ear Disease (AIED) and (2) sudden SNHL (SSNHL). Timely administration of glucocorticoids in these two conditions may result in hearing preservation. While SSNHL is usually an isolated, unilateral and single event, patients with AIED usually experience multiple episodes of rapid hearing loss in both ears. This disorder can be an isolated process affecting the ear only, or it may be part of a systemic autoimmune disorder in approximately 30% of those afflicted [1]. Further characterization of AIED has been hampered by limited access to the inner ear, and by the absence of a reliable biomarker that distinguishes AIED from patients with other forms of SNHL [2]. Thus, the incidence and prevalence of AIED is unknown, however an initial response to glucocorticoids may assist to define this clinical disorder [1], [3], [4], [5]. In patients with AIED, hearing preservation with glucocorticoids is often only maintained short term [3], [6]. Of the 60 [3] to 70% [6] of patients who are initially steroid responsive, only 14% remain so after 34 months [6]. It is unknown whether these patients who initially respond, and then become refractory to steroid therapy, have undergone a change in immune response that precludes continued response to corticosteroids. Moreover, on a molecular level, it is unknown how steroids induce a clinical response.

Medical therapy for AIED patients refractory to corticosteroids is limited. Methotrexate has been shown to be beneficial in several small series; however, in a large prospective trial, methotrexate was comparable to placebo in maintaining hearing in AIED patients [5]. In animals, suppression of TNFα by Etanercept was helpful in treating AIED, however, a recent human clinical trial failed to confirm this [7]. When medical therapy fails to restore hearing, and hearing aids are no longer beneficial, cochlear implantation is often used to restore hearing in patients with AIED who develop bilateral, severe to profound SNHL. Identification of a more effective medical therapy for AIED is contingent on developing a better understanding of the mechanism(s) that cause this disorder.

A much more common entity treated with corticosteroid therapy is sudden sensorineural hearing loss (SSNHL). A fraction of patients with SSNHL are considered to have AIED [8], as the majority of these patients are thought to have either viral or vascular pathophysiologic causes of their disease. Nonetheless, therapy for all patients with a sudden decline in hearing is similar and usually treatment entails the prompt use of corticosteroids. Evidence of an autoimmune mechanisms being important in SSNHL has been reported, and includes an increased prevalence of antibodies specific for a HSP70 (a heat shock protein), commonly found in the blood of a cohort of SSNHL patients [9], and an increased frequency of the HLA-DRB1*04 allele in SSNHL, which is associated with a poor response to glucocorticoids [10]. HLA-DRB1*04 has also been associated with other autoimmune disorders such as autoimmune thyroiditis and rheumatoid arthritis [11], [12].

Many autoantibodies to cochlear and other self-antigens in human serum have been shown to be enriched in AIED patients. Presumptive markers for AIED that has been identified include autoantibodies to: ANA (18–43%), HSP70 (22–89%) and others (recently reviewed [13]). However, no single antibody is present in all patients with this disease [2]. An increased prevalence of several autoantibodies have correlated with glucocorticoid-responsive AIED, including antibodies to both HSP70 [14] and CTCL2, recently suggested to be a biomarker for this disease [15]. HSP 70 garnered a great deal of attention because of its high specificity for AIED [14]; however its pathogenic role is controversial [16], since mice immunized with bovine HSP 70 failed to experience hearing loss [17].

Animal models of AIED have demonstrated that the inner ear is accessible from the peripheral circulation and no strict anatomic barrier exists within the cochlea. Immunocytes have been shown to traffic into the inner ear from the peripheral circulation [2], [18], and radiolabeled lymphocytes have been shown to enter the scala tympani of the cochlea [2], [18]. Furthermore, T cells from the systemic circulation have been shown to proliferate in the endolymphatic sac, and perilymph proteins of the cochlea can activate immunocytes in the endolymphatic sac [19]. These passive transfer experiments suggest that there is no anatomic barrier to trafficking lymphocytes from the systemic circulation into the inner ear.

Recent animal studies suggest that macrophages (MФ), which are part of the innate immune response, can augment adaptive T-cell responses made in AIED [20]. In these experiments, re-exposure to KLH antigen in the presence of systemic lipopolysaccharide (LPS) caused upregulation of Interleukin-1β (IL-1β) expression in the cochlea, leukocyte entry into the cochlea, and subsequent hearing loss. As anticipated, LPS induced Toll Like Receptor 4 (TLR4) signaling and augmented IL-1β expression and was associated with hearing loss. These experiments suggest that the innate immune system may participate in the development of AIED [20]. In addition, the production of IL-1β by MФ, likely causes T-cell polarization to Th1-like response that might perpetuate AIED. A critical role of pro-inflammatory CD4+, Th1-like T-cells has been shown in the cochlin peptide-murine model [21]. Interferon-gamma (IFNγ)-producing, Th1-like T cells have been identified in the peripheral blood of AIED patients [22]. Modulation of interleukin-1β levels has resulted in IFN-γ expression by T-cells in other autoimmune disorders. In murine autoimmune encephalomyelitis, blockade of the IL-1 receptor antagonist (IL-1RA) permits soluble IL-1β to bind the IL-1 receptor type 1 (IL-1R1), augmenting the development of clinical encephalomyelitis and T-cells expression of IFN-γ [23]. Thus, the absence of IL-RA expression, or other molecules that oppose the IL-1β inflammatory cascade, during an immune response, can promote the development of autoimmune disease, including AIED.

## Results

**Figure 1:**
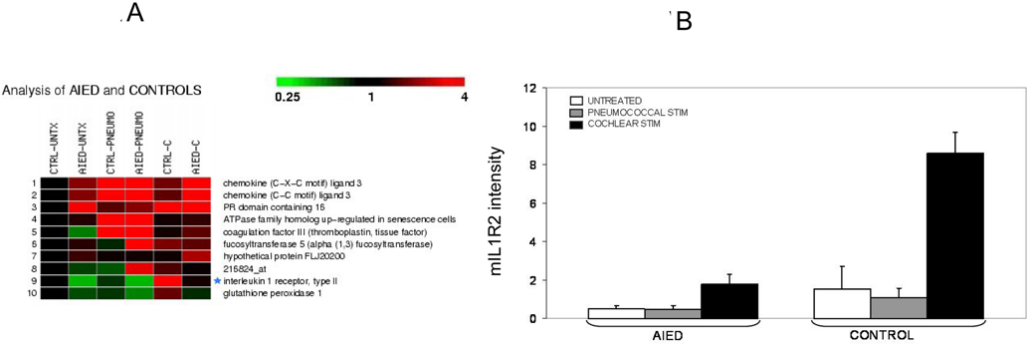
Differential expression of IL1R2 by microarray in response to autologous cochlear perilymph in patients undergoing cochlear implantation. 1A Comparison of RNA expression using the Affymetrix U133A 2.0 chip to compare cultured PBMC of controls (CTRL) and AIED patients for the following conditions: either unstimulated (UNTX), stimulated with 23-valent pneumococcal vaccine (PNEUMO) or autologous cochlear perilymph (C). Only 10 genes (panel A) were significantly different (p<0.05) when a threshold of 2 and a Benjamini & Hochberg correction was applied (Genesifter, VixXlabs). Only one of them unique to the cochlear fluid stimulated condition IL1R2 (affy ID 205403), P<0.05. Array data in 1A can be viewed at www.ncbi.nlm.nih.gov/geo/ using the user “Vambutas” with a password “daniella” to view series “GSE4277” containing 18 arrays from the six patients with “1” next to their description in table 1. 1B A comparative analysis of IL1R2 expression in these arrays is seen in panel 1B, with standard deviations shown.

We hypothesized that patients with AIED have a systemic immunologic disease manifested in the inner ear initiated by a unique cochlear antigen. Given that PBMC are capable of trafficking to the inner ear during inflammation [2], we anticipated that immunocytes that had previously encountered a cochlear antigen would respond differently to cochlear proteins compared with those that had not been previously exposed. For this reason, we compared the effect of adding autologous perilymph to peripheral blood mononuclear cells (PBMC) in patients with AIED who underwent cochlear implantation (end stage disease) compared to PBMC from patients undergoing implantation for longstanding, non-immunologic, stable, SNHL (hereafter called controls) using RNA expression microarray analysis. Patient data can be seen in table 1. Results for each patient were compared to unstimulated autologous PBMC (negative control) and pneumococcal-stimulated autologous PBMC. The rationale for the pneumococcal stimulation is that the standard care for all patients undergoing cochlear implantation is prior immunization with pneumococcal vaccine to prevent meningitis [24]. Thus, all patients were primed by vaccination with a 23-valent-pneumococcal vaccine (Pneumovax®). The majority of those with AIED also had systemic autoimmune disease. Interestingly, none of these subjects had the same autoimmune disorder, reducing the possibility of a single class II HLA allele as the mediating factor. Microarray results for AIED and control subjects were analyzed by comparing unstimulated PBMC, with autologous perilymph stimulated, and pneumococcal stimulated, PBMC (figure 1A). Analysis of the microarray data was performed by normalizing data sets by RMA and an ANOVA analysis was performed on grouped arrays by condition with a Benjamini and Hochberg correction, and a threshold of 2.0 fold change (Genesifter, VixXlabs). Of note, only 10 genes were differentially expressed in these experiments when the threshold was set at 2 and a Benjamini & Hochberg correction applied (p<0.05). Of those 10 genes, only one, the interleukin-1 receptor type 2 (IL1R2) (affymetrix ID 205403, nt 1104–1485 (long membrane bound form of IL1R2 (mIL1R2)), was differentially expressed when autologous perilymph was added to PBMC of control subjects, as compared with AIED subjects (Figure 1A, 1B). Analysis of IL-1RI, IL-1β, IL-1RA, heat shock proteins (HSP) and TNF-α all failed to show differential expression (not shown).

**Table 1:** Clinical History of AIED and control patients undergoing cochlear implantation.

Patient	Status	Autoimmune History	Hearing History	PTA & HINT	Serology
40 y/o female^1,2^	**Control** (birth anoxia?)	None	Congenital loss – non progressive	PTA 100 dB; HINT 60 dB quiet: 0%	NP*
49 y/o male^1,2^	**Control**	None	40+ years loss	PTA 100 dB; HINT 50 dB quiet: 10%	NP*
79 y/o female^1,3^	**Control** (LVA)	None	70+ years LVA	PTA 110; HINT 55 dB quiet: 0%;	NP*
1.5 y/o female^3^	**Control** (Cx 26)	None	Congenital – stable	PTA: NR at 102 dB by ABR; IT-MAIS: 12%	NP*
36 y/o female^1,2^	**AIED**, progressive loss	Rheumatoid Arthritis	29 years, periods of rapid loss	PTA 120 dB; HINT: 55 dB quiet: 0%	P0, Rheumatoid factor IgM (14.2) & IgG (25.3), immune complex, ANA 1∶1280 (HEp-2), IgM phospholipids (10.9), immune complex (26.1)
56 y/o male^1,2^	**AIED**, rapid loss	Type I IDDM	2 years, rapid loss	PTA 120; HINT 70 dB quiet: 0%	ANA 1∶1280 (mouse kidney), ANA 1∶320 (HEp-2),weakly positive IgM phospholipid Ab (12.1)
58 y/o female^1,2,3^	**AIED**, rapid loss	Hashimoto's thyroiditis	4 years, rapid loss	PTA 110 dB; HINT at 50 dB quiet: 0%	Borderline for immune complex (21.4), weakly positive IgM phospholipids (11.7), borderline rheumatoid factor IgG (20.8)
51 y/o female^2,3^	**AIED**, progressive loss	None	Sudden loss left ear 10 years prior, sudden loss right ear <6 months	PTA 80 dB; HINT 65 dB quiet: 19%	68 kD positive
3 y/o male^3^	**AIED**, b/l rapid SNHL	None	6 months, rapid loss	PTA 120 dB; IT-MAIS 8%	Negative

**Figure 2:**
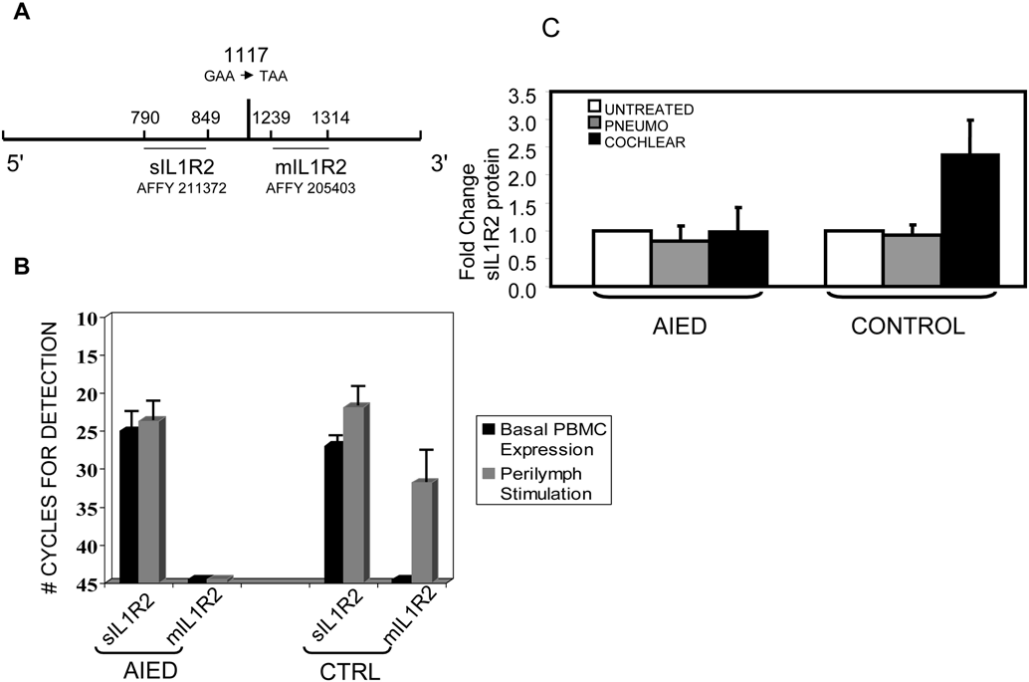
Expression of IL1R2 in cochlear implant recipients. 2A Diagram of IL1R2 mRNA depicting regions detected by microarray probes and Q-RT-PCR primers. At position 1117, an alternate splice site has been described [25] that induces a stop codon. 2B Q-RT-PCR for 3 control subjects and 5 AIED subjects comparing autologous perilymph stimulated to unstimulated PBMC for both the shorter soluble IL1R2 (sIL1R2) and the longer membrane bound form of Il1R2 (mIL1R2). Results were dramatically different and statistically significant for mIL1R2 (as seen with an asterix (*)), using a Mann Whitney test (p = 0.03) (in stat). 2C ELISA for soluble IL-1R2 (sIL1R2). Supernatants from 16-hour PBMC cultures from 3 AIED patients and 2 controls were used to determine the level of soluble receptor. Stimulus conditions were no stimulus (white bar), pneumococcal stimulus (grey bar) or autologous cochlear perilymph (black bar). Relative amounts were set at one for the unstimulated condition as this data set included 2 children (one AIED, one control). sIL-1R2 levels are less in children however, a similar patterns of expression were observed. Standard deviations are shown.

PTA: Pure tone average of the audiogram at 500, 1000, 2000 and 4000 Hz, (although 4000 Hz is not traditionally included, this information is important to speech perception for cochlear implantees and therefore PTA represents these four frequencies for these patients). NP: Not Performed: control subjects did not have serology for AIED performed as there was no clinical indication for such studies, HINT: Hearing in Noise Test; IT-MAIS: Infant-Toddler Meaningful Auditory Integration Scale, LVA: Large Vestibular Aqueduct; Cx26: connexin 26 gene mutation. Superscript numbers in column 1 refer to inclusion of the patient in data sets for the below figures: 1: included in analysis for figure 1; 2b & c: included in analysis for figure 2b or 2c. In one patient, all RNA was used for microarray analysis (79 y/o female, control).

### IL1R2 Expression

To confirm our microarray results, we used a quantitative real time polymerase chain reaction (Q-RT-PCR). IL1R2 exists in two forms: membrane-bound (mIL1R2) and soluble (sIL1R2). The RNA primers used to detect the membrane bound, long form of the IL1R2 message were derived from the coding region of the IL1R2 gene present on the Affymetrix gene array chip (figure 2A). The shorter soluble form is made by alternative splicing [25], or by enzymatic cleavage of the membrane bound protein by either a metalloproteinase, aminopeptidase or elastase [26], [27], [28], however, the shed IL1R2 size varies with a metalloproteinase producing a 45 kD product, and aminopeptidase and elastase producing a 60 kD product [26]. Interestingly, both AIED patients and controls make transcripts for the shorter form, although at slightly lower levels in AIED subjects compared with controls (affymetrix ID 211372, nt 635–1084) (figure 2B). RNA for the longer, membrane bound form, is not expressed in perilymph stimulated PBMC from AIED patients (no transcript detected at 45 cycles); however, in perilymph stimulated control PBMC, mIL1R2 RNA is strongly expressed (figure 2B). The difference was statistically significant using a Mann Whitney test (p = 0.03) (in stat).Notably, the inhibitory function of IL1R2 is selectively attributed to this long, membrane bound form [29].

Soluble IL1R2 levels, which reflect of the shorter IL1R2 message (affymetrix 211372), were reduced in AIED patients in culture supernatants of PBMC stimulated with perilymph measured by ELISA (figure 2C). This finding parallels our microarray results for Affymetrix probe 211372 (not shown) and our Q-RT-PCR expression of sIL1R2 in AIED and control subjects (figure 2B). Interestingly, at least for the AIED cochlear implant recipients, there did not appear to be increased sIL1R2 as the mechanism for IL1R2 reduction compared to controls.

### IL1R2 splicing correlates with clinical steroid responsiveness

**Figure 3:**
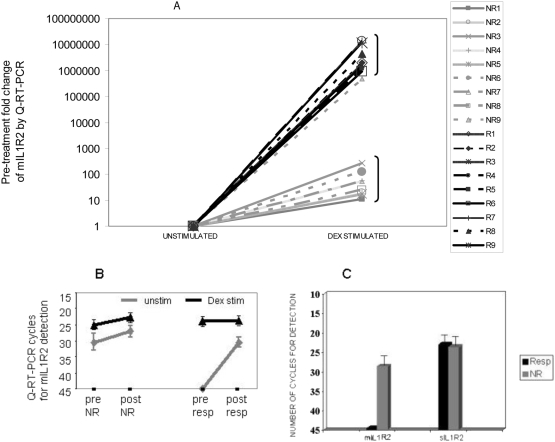
Pre-treatment induced expression of mIL1R2 in response to dexamethasone correlates with post-treatment clinical hearing restoration. 3A 18 patients with presumptive AIED and sudden declines in hearing were enrolled, blood drawn prior to treatment, and treated with 60 mg prednisone/day×7 days and then tapered. Pre-treatment PBMC were divided and incubated with either no stimulus or dexamethasone (8 µg/ml is shown). IL1R2 mRNA was measured by Q-RT-PCR. Post-treatment audiograms were obtained between 7–14 days later. Audiometric improvement (Δ) was measured as the average of 250, 500, 1,2,& 4 kHz. Responders (R) all returned to prior baseline hearing. Pre-treatment levels of mIL-1R2 Q-RT-PCR (expressed as fold change over baseline) predicted steroid responsiveness in AIED (P<0.0001, Mann Whitney test). 3B Pre and post treatment mIL1R2 levels in a subset of clinical responders and non-responders. Pre-treatment responders have no basal mIL1R2 expression in PBMC (unstim) at 45 cycles (in replicate samples), compared with non-responders that have substantially higher basal levels. Although PBMC from both responders and non-responders demonstrate increased mIL1R2 RNA expression in response to in vitro dexamethasone stimulation (dex stim), responders are exquisitely sensitive. The concentration of dexamethasone shown for the PBMC stimulation experiments was 20 ug/ml, however, 4 ug/ml and 8 ug/ml produced identical results, suggesting maximal stimulation even at 4 ug/ml. 3C Despite clear differences in mIL1R2 expression patterns, sIL1R2 expression is differences between responders and non-responders are minimal suggesting alternate splicing in clinical responders.

Only 60% of patients studied by the AIED study group were steroid responsive [3]. To date, it is unknown why some AIED patients respond while others do not, although presence of certain antibodies have been correlated with steroid responsiveness [14], [15]. We hypothesized that robust expression of mIL1R2, in response to corticosteroids may be indicative of a clinical response. To test the hypothesis, we enrolled patients with acute deterioration of their hearing that met the same criteria that the AIED study group used [3]. Blood for PBMC isolation was drawn immediately prior to oral steroid therapy (60 mg of prednisone daily for a minimum of 7 days with a variable taper thereafter). A repeat audiogram was performed between 7–10 days post initiation of treatment, and in a subset of patients, blood for PBMC isolation was again drawn. Table 2 describes the eighteen patients enrolled in this study. The average hearing recovery at all frequencies tested is shown in Table 2. All responders returned to their prior baseline hearing thresholds at the end of the glucocorticoid therapy. Non-responders were defined as those patients that showed less than 5 dB improvement at all frequencies tested, or further decline in pure tone thresholds. In these studies, we observed that pre-treatment expression of mIL1R2 mRNA by PBMC in response to dexamethasone stimulation *in vitro*, correlated with clinical responsiveness to oral steroid therapy. Corticosteroid responders had absent pre-treatment, basal mIL1R2 expression; however, they dramatically augmented mIL1R2 expression in response to dexamethasone stimulation, p<0.0001 (figure 3A), using a Mann Whitney test. Although the mean and standard deviations are large in the groups largely due to the steroid non-responsive patient that clustered with the steroid responsive group, the median fold change of the non-responsive group was 47 as compared to the median of the responsive group was 2.37×10^6^. If we set a threshold for clinical steroid responsiveness to be a greater than 500 fold induction of mIL1R2 in vitro, then the sensitivity of this test is 100% with a confidence interval of 71.7 to 100%. The specificity would be 88.9% with a confidence interval of 51.8–99.7%. The dramatic difference in the pre-treatment fold change of the corticosteroid responders was inversely proportional to basal mIL1R2 expression by unstimulated PBMC (45 cycles, S.D. 0.0 in replicate samples), (denoted “unstim” in figure 3B), compared to corticosteroid non-responders (28.48 cycles, S.D.±3.34) (Figure 3B). Post-treatment basal expression of mIL1R2 in steroid responders was detected at a much lower cycle number by Q-RT-PCR than pre-treatment (figure 3B). Pre-treatment basal mIL1R2 expression in glucocorticoid-responders dramatically increased from no expression at 45 cycles to detectable expression at 30 cycles post-treatment, compared with 30 cycles to 27 cycles post-treatment in non-responders suggesting that the glucocorticoid responsiveness correlated with induction of mIL1R2 RNA expression (figure 3B). Furthermore, as seen with the cochlear implant patients, despite absent mIL1R2 expression, sIL1R2 expression was observed at reasonable levels (figure 3C). Interestingly, the steroid refractory patients demonstrated high mIL1R2 expression in absence of stimulation prior to treatment, with no major change in expression in response to dexamethasone (figure 3B, denoted “dex stim”). This expression pattern suggests some endogenous stimulation of mIL1R2 expression.

**Table 2:** Clinical History of patients treated with prednisone.

PATIENT	PRIOR TX?	PMH	SEROLOGY	MRI	AFFECT EAR	250 Hz	500 Hz	1000 Hz	2000 Hz	4000 Hz	AVG Improve
56WM (NR1)	YES	NC	NA	NEG	PRETX	35	45	60	105	120	−1 dB
					POSTTX	40	45	60	105	120	
42 WF^B^ (NR2)	YES	Hypo-thyroid	ANA 1∶320, Immune complex +	NEG	PRETX	50	75	90	95	95	−1 dB
					POSTTX	55	75	95	95	90	
61HF^B^ (NR3)	YES	NC	NA	NEG	PRETX	65	60	40	35	45	−3 dB
					POSTTX	70	60	50	35	45	
57 WM^B^ (NR4)	YES	Brother W/Crohns	ANA1∶2560, P0 pos	NEG	PRETX	55	60	75	65	80	−19 dB
					POSTTX	60	70	75	70	75	
45WM (NR 5)	YES	NC	RF borderline +	NEG	PRETX	60	60	65	65	70	+2 dB (NS)
					POSTTX	60	60	70	60	60	
62 WF (NR6)	YES	NC	Weak+68 kD Borderline typeII collagen	NEG	PRETX	10	10	10	10	40	−9 dB
					POSTTX	15	15	10	10	55	
60 WF (NR7)	YES	Pulm collag vasc disease	P0 pos	NEG	PRETX	60	60	60	55	75	−1 dB
					POSTTX	65	60	65	55	70	
WM (NR8)	YES	Father w/Auto-immune disease req. steroids	Neg	NEG	PRETX	50	55	65	65	60	0 dB
					POSTTX	50	55	65	60	65	
46 WF (NR9)	NO	Multiple Sclerosis	NA	NEG	PRETX	95	90	95	120	120	−9 dB
					POSTTX	100	110	115	120	120	
56 WF^B^ (R1)	YES	Daughter W/IDDM	ANA 1∶640, Phospholipid IgG & IgM pos	NEG	PRETX	55	60	60	40	40	**+37 dB**
					POSTTX	15	20	15	5	15	
54 WF^B^ (R2)	YES	Hashimotos Thyroiditis	ANA 1∶160, 68 kD pos	NEG	PRETX	105	115	100	100	100	**+39 dB**
					POSTTX	40	60	70	80	75	
39WM^B^ (R3)	YES	NC	NA	NEG	PRETX	40	45	35	15	30	**+15 dB**
					POSTTX	25	15	5	5	40	
69 WF (R4)	YES	Crohn's Disease	NA	NEG	POSTTX	40	35	30	30	20	**+6 dB**
					POSTTX	20	35	25	30	15	
59 WF (R5)	YES	Fibromyalgia	NA	NEG	PRETX	105	105	105	100	95	**+9 dB**
					POSTTX	105	90	90	90	90	
53 WM (R6)	YES	Psoriasis	NA	NEG	PRETX	50	55	45	25	25	**+17 dB**
					POSTTX	35	30	15	10	35	
46 WM (R7)	YES	NC	NA	NEG	PRETX	55	60	30	10	60	**+11 dB**
					POSTTX	50	30	15	5	60	
79 WF (R8)	YES	NC	NA	NEG	PRETX	50	40	40	55	80	**+11 dB**
					POSTTX	25	25	40	55	70	
37 WF (R9)	YES	NC	Neg	NEG	PRETX	20	30	45	60	75	**+19 dB**
					POSTTX	10	15	20	35	55	

“Prior tx?”: although all had prior rapid declines in hearing requiring prednisone therapy, none were treated in a 3 month period prior to enrollment. Several patients had occasional vestibular symptoms, however, these symptoms did not coincide with periods of hearing fluctuation. Serology was performed at Immco Diagnostics. NA = not available: serologic testing at Immco could not be performed as these tests were not covered b y the patient's insurance carrier. Audiometric change shown represents the change between the average of the post-treatment pure tone thresholds from the pre-treatment pure tone average threshold. NS = not significant: average post-treatment hearing changes of less than 5 dB are not felt to represent significant changes. All declines in hearing are reported as 0 dB. NR = clinical non-responder; R = clinical responder.

It is paradoxical that AIED cochlear implant recipients demonstrated absent basal expression of mIL1R2, yet steroid refractory AIED patients exhibit high levels of mIL1R2 in absence of in vitro stimulation. Steroid-refractory patients with high basal expression of mIL1R2 may represent a different disorder. Alternatively, steroid-refractory patients have a different inflammatory microenvironment that overwhelms the effects of mIL1R2-induced expression. The absent basal expression of mIL1R2 in AIED cochlear implant recipients may reflect the absence of acute inflammation at the time of implantation. Our results demonstrate that low basal and induced expression of mIL1R2 in AIED during periods of inflammation and rapid hearing decline correlated with the clinical response to glucocorticoids.

## Discussion

IL1R2 is known to be a molecular decoy that sequesters IL-1β, and blocks the initiation of downstream signaling, thereby preventing inflammation (as reviewed [30], [31]. The IL1R2 protein is expressed on B cells, monocytes/macrophages and neutrophils [30], and may be expressed in response to many stimuli, including oxygen radicals, IL-4, IL-13, and glucocorticoids [30], possibly explaining the ability of steroids to ameliorate AIED. Release of the soluble IL1R2 (sIL1R2) may also be induced by various stimuli, although the major inhibitory function of IL1R2 has been ascribed to the membrane bound form [29]. The induction of IL1R2 by IL-4 [32] certainly suggests that the maintenance of a T_H_2-like phenotype is important in controlling inner ear disease, whereas expression of IFNγ, that fosters a T_H_1-like response, has been observed in AIED patients [22], and blocks the expression of IL1R2 [33]. IL1R2 is an important mechanism that prevents IL-1β-mediated inflammation at sites where inflammation is poorly tolerated. In the brain, IL1R2 is preferentially expressed compared to IL-1R1, the cognate IL-1β receptor [34]. Restoring IL1R2 expression in IL1R2 deficient cells resulted in significant cellular protection from IL-1β mediated damage [35]. Treatment of mice with IL1R2 expressing cells reduced inflammation in collagen-induced arthritis [36], demonstrating that IL1R2 may be beneficial in diseases where IL-1β is dominant and endogenous counteracting antagonists are absent.

In steroid responsive patients, sIL1R2 RNA was detected in unstimulated PBMC, however, the long mIL1R2 transcript could not be detected. In the presence of dexamethasone, the long transcript could readily be detected. LPS stimulation of monocytes results in reduced mRNA synthesis of IL1R2 [28]. Although transcriptional induction of IL1R2 from dexamethasone stimulation of monocytes has been studied extensively by Northern analysis [37], [38], [39], alternate splicing has not been reported, perhaps because these studies were performed in a limited number of control subjects or small molecular weight shifts induced by alternate splicing near the 3′ end may not have been detected in these studies. Dexamethasone has however, been reported to induce differential splicing in other genes [40]. Furthermore, alternate splicing of critical regulatory genes has been identified to associate with risk of other autoimmune diseases, such as alternate splicing of BANK1 in Systemic Lupus Erythematosus [41]. Investigation into whether genetic polymorphisms affect the splicing of the IL1R2 message is underway in our laboratory.

Although it has been shown that no anatomic barrier exists preventing leukocyte trafficking to the inner ear [2], [18], the robust expression of mIL1R2 in control cochlear implant subjects, and AIED patients who responded to glucocorticoids suggests that the inner ear likely is at least a partially functionally immunoprivileged site. We hypothesize that AIED patients who experience a break in tolerance and fail to express IL1R2 in response to antigenic stimuli, have unopposed IL-1β expression that leads to hearing loss. Unopposed expression of IL-1β has been shown to induce sensorineural deafness in patients with Muckle-Wells syndrome, an autoinflammatory disorder. In these patients, restoration of hearing was observed following anakinra therapy, an IL-1 receptor antagonist [42], [43], with normalization of IL-1β serum levels [43].

We further hypothesized that the expression of IL1R2, in response to various stimuli, likely represents an innate immune response by macrophages. Macrophages are the predominant producer of IL1R2 [37], [39]. Macrophage ingress into the cochlea has been demonstrated in traumatic injury: perhaps best characterized during periods of acoustic-induced trauma [44]. Furthermore, during acoustic trauma, pro-inflammatory cytokines, including IL-1β are expressed [45]. Similarly, IL-1β expression was observed in response to LPS in an animal model of AIED [20]. Reduction of mononuclear cell secretion of IL-1β was observed following anakinra therapy and correlated with hearing restoration in a patient with Muckle-Wells syndrome [43]. We hypothesize that as the AIED progresses, an increased production of IFN-γ blocks expression of IL1R2 in the cochlea. Preliminary flow cytometry experiments demonstrate induction of IL1R2 surface expression in response to dexamethasone occurs in blood monocytes in these patients (not shown). We are also examining the perilymph of these patients to ascertain whether it is the cytokine microenvironment, or the presence of a unique protein or autoantigen found in perilymph that is inducing differential IL1R2 expression in perilymph from AIED and controls.

To date, ability to identify glucocorticoid responsive AIED patients prior to treatment has been limited to a correlation with seropositivity for either HSP70 or CTCL2 [14], [15]. Thus, identifying mIL1R2 levels that predict steroid responsiveness early on in disease may help better phenotype those with AIED, and those who will benefit from this therapy thereby avoiding the complications associated with this treatment in patients not expected to respond.

The observations we describe suggest that mILR2 expression may be an important mechanism involved in glucocorticoid responsiveness in AIED; however, given that steroids control inflammation by multiple mechanisms, it is probable that other pathways contribute to the observed response. Clinical therapies that alter IL-1β expression have not been used in AIED patients.

## Methods

### Patient Recruitment

#### Ethics Statement

These studies were approved by the North Shore-LIJ Health System IRB. All patients signed written consent for inclusion into these studies. In the case of the two children, their parents provided written consent. Assent was not obtained from the children due to their young age.

#### Cochlear Implant Subjects

All patients met current criteria for cochlear implantation and had a bilateral profound SNHL. All patients with AIED had periods of rapid hearing loss and were no longer steroid responsive. All adult controls had stable SNHL for over 30 years of clear, non-immunologic origin. A total of 9 patients were recruited (7 adults 2 children) representing 4 controls and 5 with AIED, and their clinical demographics shown in table 1.

At the time of cochlear implant surgery; blood was drawn to isolate PBMC. Perilymph fluid was harvested from the cochleostomy in a bloodless field. In order to control for a cochlear perilymph specific response, we compared unstimulated PBMC, to cochlear perilymph stimulated PBMC, and pneumococcal stimulated PBMC, from both AIED and control patients. Three microarrays were performed per study subject: 1) perilymph+autologous PBMC, 2) pneumococcal antigen+autologous PBMC, and 3) PBMC alone and administration of pneumococcal vaccine 2 weeks prior to cochlear implant surgery, considered to be standard of care prior to cochlear implantation, served as a positive control.

### Prospectively Enrolled, Steroid Treated Subjects

Eighteen patients with AIED who had not undergone cochlear implantation were studied in a separate IRB approved protocol (Table 2). They all had a clinical history suggestive of AIED, and met criteria defined for AIED trials [3]. At the time of enrollment, they all demonstrated sudden declines in their hearing and were treated with 60 mg of prednisone daily for 7 days with a variable taper thereafter. The responders were defined as a 5 dB or greater average improvement at 250, 500, 1000, 2000 and 4000 Hz. All patients recruited must have had prior audiograms demonstrating their baseline-hearing threshold. Furthermore, SNHL of greater than 30 dB at one or more frequencies in *both* ears with evidence of active deterioration (elevated threshold) in at least one ear of 15 dB at one frequency (excluding 250 Hz as a sole indicator), or 10 dB at 2+ frequencies developing in >3 days but <90 days [3], [16]. If the hearing loss evolved in less than 3 days, prior similar hearing declines must have occurred or the patient must have a systemic autoimmune disorder AND the patient must meet the audiology criteria outlined above. All patients with retrocochlear (vestibular schwannoma or other internal auditory canal s) pathology), patients who received prednisone or other immunosuppressive therapy within 3 months, or patients with vestibular symptoms coinciding with periods of hearing fluctuation [3] were all excluded from this study. All patients received a minimum of 60 mg of prednisone daily for 7 days with a variable taper thereafter.

#### Microarray Analysis

PBMC were isolated over a Ficoll-hypaque gradient, divided into 5 ml cultures (2×10^6^ cells/ml) and cultured in RPMI+10%FCS for 16 hours at 37°C with 5% CO_2_ with one of 3 stimuli (1) without antigenic stimulus (untx), (2) 100 µl pneumococcal vaccine [46] as a positive control (pneumo) or (3) with 15 µl of autologous cochlear perilymph (c). RNA was isolated using an affinity spin column (Qiagen) and 5 µg reverse transcribed into double stranded cDNA (Invitrogen) incorporating a T7 RNA polymerase promoter. The cDNA was phenol extracted, ethanol precipitated and biotinlyated cRNA generated by in vitro transcription (ENZO). cRNA was purified on a spin column (Qiagen), quantitated, and 20 µg fragmented and hybridized to the Affymetrix HG U133A 2.0 array (Affymetrix). Data sets were normalized using RMA and an ANOVA analysis was performed on grouped arrays by condition with a Benjamini and Hochberg correction, and a threshold of 2.0 fold change (Genesifter, VixXlabs). Array data in 1A can be viewed at www.ncbi.nlm.nih.gov/geo/ using the user “Vambutas” with a password “daniella” to view series “GSE4277” containing 18 arrays from the six patients with “1” next to their description in table 1. The array data is MIAME compliant. Upon publication, this data will be publicly released, with the password removed.

#### Q-RT-PCR

Quantitative Real Time RT-PCR (Q-RT-PCR) was performed on PBMC from patients using TaqMan chemistry. The relative abundance of IL1R2 mRNA for 2 membrane bound (mIL1R2) and the soluble (sIL1R2) coding regions associated with the 2 affymetrix probes sets 211372 (sIL1R2) and 205403 (mIL1R2) were compared to β-actin was determined using the Eurogentec RTqPCR mastermix (Eurogentec, Belgium) and ABI PRISM 7700 Sequence Detection System. Membrane bound IL1R2, as reflected by affymetrix probe set 205403 was detected by primers nt 1239–1258, 1295–1314, and taqman probe 1271–1278, whereas the shorter soluble form (sIL1R2) reflected by the affymetrix probe 211372 and primers nt 790–830–849, and taqman probe 820–827. The alternative splice site described by Liu changing GAA to TAA, introducing a stop codon, occurs at nt 1118 [25]. These primers were added at final concentration of 200 nM and 100 nM respectively to 50 ng of total RNA. The conditions were 48°C for 30′, 95° C for 10′ and 45 cycles of 95°C for 15″ and 60°C for 1′. Data was analyzed using Sequence Detection System software version 1.9.1. Results were expressed as Ct (Threshold cycle) values, which is inversely proportional to the starting template copy number. Relative abundance of IL1R2 in cochlear fluid stimulated cells was calculated compared to untreated control samples using *delta delta Ct method* (User Bulletin #2, ABI). In all prospectively enrolled, steroid treated patients, the unstimulated condition is the average of 2 independent PBMC cultures (SD for responders was 0.0, and SD for non-responders was 3.34 cycles).

#### ELISA Analysis

ELISA for soluble IL-1R2 (sIL-1-R2). Supernatants from 16-hour PBMC cultures from 3 AIED patients and 2 controls were used to determine the level of soluble receptor (See table 1 for inclusion). ELISA was performed according to manufacturers' instructions (R&D Systems). Stimulus conditions were either pneumococcal stimulus as used above or autologous cochlear perilymph and compared to unstimulated PBMC. A large number of duplicate samples were run to ensure accuracy.

## References

[1] Ruckenstein MJ (2004). Autoimmune inner ear disease.. Curr Opin Otolaryngol Head Neck Surg.

[2] Ryan AF, Harris JP, Keithley EM (2002). Immune-mediated hearing loss: basic mechanisms and options for therapy.. Acta Otolaryngol.

[3] Niparko JK, Wang NY, Rauch SD, Russell GB, Espeland MA (2005). Serial audiometry in a clinical trial of AIED treatment.. Otol Neurotol.

[4] McCabe BF (1979). Autoimmune sensorineural hearing loss.. Ann Otol Rhinol Laryngol.

[5] Harris JP, Weissman MH, Derebery JM, Espeland MA, Gantz BJ (2003). Treatment of corticosteroid-responsive autoimmune inner ear disease with methotrexate: a randomized controlled trial.. JAMA.

[6] Broughton SS, Meyerhoff WE, Cohen SB (2004). Immune mediated inner ear disease: 10-year experience.. Semin Arthritis Rheum.

[7] Cohen S, Shoup A, Weisman MH, Harris J (2005). Etanercept treatment for autoimmune inner ear disease: results of a pilot placebo-controlled study.. Otol Neurotol.

[8] Berrocal JR, Ramirez-Camacho R (2002). Sudden sensorineural hearing loss: supporting the immunologic theory.. Ann Otol Rhinol Laryngol.

[9] Park SN, Yeo SW, Park KH (2006). Serum heat shock protein 70 and its correlation with clinical characteristics in patients with sudden sensorineural hearing loss.. Laryngoscope.

[10] Amor-Dorado JC, Paco L, Martin J, Lopez-Nevot MA, Gonzalez-Gay MA (2005). Human leukocyte antigen-DQB1 and -DRB1 associations in patients with idiopathic sudden sensorineural hearing loss from a defined population of Northwest Spain2005.. Acta Otolaryngol.

[11] Zantut-Wittmann DE, Persoli L, Tambascia MA, Fischer E, Franco Maldonado D (2004). HLA-DRB1*04 and HLA-DQB1*03 association with the atrophic but not with the goitrous form of chronic autoimmune thyroiditis in a Brazilian population.. Horm Metab Res.

[12] Alsaeid K, Alawadhi A, Al-Saeed O, Haider MZ (2006). Human leukocyte antigen DRB1*04 is associated with rheumatoid arthritis in Kuwaiti patients.. Joint Bone Spine.

[13] Agrup C, Luxon LM (2006). Immune mediated inner-ear disorders in neuro-otology.. Cur Opin Neurol.

[14] Moscicki RA, San Martin JE, Quintero CH, Rausch SD, Nadol JB (1994). Serum antibody to inner ear proteins in patients with progressive hearing loss. Correlation with disease activity and response to corticosteroid treatment.. JAMA.

[15] Zeitoun H, Beckman JG, Arts HA, Lansford CD, Lee DS (2005). Corticosteroid response and supporting cell antibody in autoimmune hearing loss.. Arch Otolaryngol Head Neck Surg.

[16] Yeom K, Gray J, Nair TS, Arts HA, Telian SA (2003). Antibodies to HSP-70 in normal donors and autoimmune hearing loss patients.. Laryngoscope.

[17] Trune DR, Kempton JB, Mitchell CR, Hefeneider SH (1998). Failure of elevated heat shock protein 70 antibodies to alter cochlear function in mice.. Hear Res.

[18] Gloddek B, Arnold W (2002). Clinical and experimental studies of autoimmune inner ear disease.. Acta Otolaryngol.

[19] Iwai HTK, Tomoda K, Sugiura K, Inaba M, Ikehara S (1999). T cells infiltrating from the systemic circulation proliferate in the endolymphatic sac.. Ann Otol Rhinol Laryngol.

[20] Hashimoto S, Billings P, Harris JP, Firestein GS, Keithley EM (2005). Innate Immunity Contributes to Cochlear Adaptive Immune Responses.. Audiology & Neurotology.

[21] Solares CA, Edling AE, Johnson JM, Baek MJ, Hirose K (2004). Murine autoimmune hearing loss mediated by CD4+ T cells specific for inner ear peptides.. J Clin Invest.

[22] Lorenz RR, Solares CA, Williams P, Sikora J, Pelfrey CM (2002). Interferon-gamma production to inner ear antigens by T cells from patients with autoimmune sensorineural hearing loss.. J Neuroimmunol.

[23] Matsuki T, Nakae S, Sudo K, Horai R, Iwakura Y (2006). Abnormal T cell activation caused by the imbalance of IL-1/IL-1R antagonist system is responsible for the development of experimental autoimmune encephalomyelitis.. Int Immunol.

[24] Reefhuis J, Honein MA, Whitney CG, Chamany S, Mann EA (2003). Risk of bacterial meningitis in children with cochlear implants.. NEJM.

[25] Liu C, Hart RP, Liu XJ, Clevenger W, Maki RA (1996). Cloning and characterization of an alternativly processed human type II interleukin-1 receptor mRNA.. J Biol Chem.

[26] Orlando S, Sironi M, Bianchi G, Drummond AH, Boraschi D (1997). Role of metalloproteases in the release of the IL-1 type 2 decoy receptor.. J Biol Chem.

[27] Cui X, Rouhani FN, Hawani F, Levine SJ (2003). Shedding of the type II IL-1 decoy receptor requires a functional aminopeptidase, aminopeptidase regulator of TNF receptor type 1 shedding.. J Immunol.

[28] Penton-Rol G, Orlando S, Polentarutti N, Bernasconi S, Muzio M (1999). Bacterial lipopolysaccharide causes rapid shedding, followed by inhibition of mRNA expression, of the IL-1 type II receptor, with concommitant up-regulation of the type I receptor and induction of incompletely spliced transcripts.. J Immunol.

[29] Neumann D, Kollewe C, Martin MU, Boraschi D (2000). The membrane bound form of the type II IL-1 receptor accounts for inhibitiory function.. J Immunol.

[30] Mantovani A, Muzio M, Ghezzi P, Colotta C, Introna M (1998). Regulation of Inhibitory Pathways of the Interleukin-1 System.. Ann NY Acad Sci.

[31] Mantovani A, Bonecchi R, Martinez FO, Galliera E, Perrier P (2003). Tuning of innate immunity and polarized responses by decoy receptors.. Int Arch Allergy Immunol.

[32] Coletta F, Re F, Muzio M, Bertini R, Polentarutti N (1993). Interleukin-1 type 2 receptor: a decoy target for IL-1 that is regulated by IL-4.. Science.

[33] Dickensheets HL, Donnelly RP (1997). IFN-gamma and IL-10 inhibit induction of the IL-1 receptor type I and II gene expression by IL-4 and IL-13 in human monocytes.. J Immunol.

[34] Docagne F, Campbell SJ, Bristow AF, Poole S, Vigues S (2005). Differential regulation of type I and type 2 interleukin-1 receptors in focal brain inflammation.. Eur J Neurosci.

[35] Bessis N, Guery L, Mantovani A, Vecchi A, Sims JE (2000). The type 2 decoy receptor of IL-1 inhibits murine collagen-induced arthritis.. Eur J Immunol.

[36] Attur MG, Dave MN, Leung MY, Cipolletta C, Meseck M (2002). Functional genomic analysis of type 2 IL-1beta decoy receptor: potential for gene therapy in human arthritis and inflammation.. J Immunol.

[37] Colotta F, Saccani S, Giri JG, Dower SK, Sims JE (1996). Regulated expression and release of the IL-1 decoy receptor in human mononuclear phagocytes.. J Immunol.

[38] Re F, Muzio M, De Rossi M, Polentarutti N, Giri JG (1994). They type II “Receptor” as a decoy target for interleukin 1 in polymorphnuclear leukocytes: characterization of induction by dexamethasone and ligand binding properties of the released decoy receptor.. J Exp Med.

[39] Brown EA, Dare HA, Marsh CB, Wewers MD (1996). The combination of endotoxin and dexamethasone induces type 2 interleukin I receptor (IL-1R2) in monocytes: a comparison to interleukin 1 beta (IL-1 beta) and interleukin 1 receptor antagonsist (IL-1ra).. Cytokine.

[40] Auboeuf D, Honig A, Berget SM, O'Malley BW (2002). Coordinate regulation of transcription and splicing by steroid receptor coregulators.. Science.

[41] Kozyrev SV, Abelson AK, Wojcik J, Zaghlool A, Linga Reddy MV (2008). Functional variants in the B-cell gene BANK1 are associated with systemic lupus erythematosus.. Nature Genetics.

[42] Mirault T, Launay D, Cuisset L, Hachulla E, Lambert M (2006). Recopvery from deafness in a patient with Muckle-Wells syndrome treated with anakinra.. Arthritis Rheum.

[43] Yamazaki T, Masumoto J, Agematsu K, Sawai N, Kobayashi S (2008). Anakinra improves sensory deafness in a japanese patient with Muckle-Wells syndrome, possibly inhibiting the cryopyrin inflammasome.. Arthritis Rheum.

[44] Hirose K, Discolo CM, Keasler JR, Ransohoff R (2005). Mononuclear phagocytes migrate into the murine cochlea after acoustic trauma.. J Comp Neurol.

[45] Fujioka M, Kanzaki S, Okano HJ, Masuda M, Ogawa K (2006). Proinflammatory cytokines expression in noise-induced damaged cochlea.. J Neurosci Res.

[46] Wuorimaa T, Kayhty H, Eskola J, Bloigu A, Leroy O, Surcel HM (2001). Activation of cell mediated immunity following immunization with pneumococcal conjugate or polysaccharide vaccine.. Scand J Immunol.

